# Toward automation of initial chart check for photon/electron EBRT: the clinical implementation of new AAPM task group reports and automation techniques

**DOI:** 10.1002/acm2.13200

**Published:** 2021-03-11

**Authors:** Huijun Xu, Baoshe Zhang, Mariana Guerrero, Sung‐Woo Lee, Narottam Lamichhane, Shifeng Chen, Byongyong Yi

**Affiliations:** ^1^ University of Maryland School of Medicine Baltimore MD USA

**Keywords:** automation, initial chart check, TG‐275, TG‐315

## Abstract

**Purpose:**

The recently published AAPM TG‐275 and the public review version of TG‐315 list new recommendations for comprehensive and minimum physics initial chart checks, respectively. This article addresses the potential development and benefit of initial chart check automation when these recommendations are implemented for clinical photon/electron EBRT.

**Methods:**

Eight board‐certified physicists with 2–20 years of clinical experience performed initial chart checks using checklists from TG‐275 and TG‐315. Manual check times were estimated for three types of plans (IMRT/VMAT, 3D, and 2D) and for prostate, whole pelvis, lung, breast, head and neck, and brain cancers. An expert development team of three physicists re‐evaluated the automation feasibility of TG‐275 checklist based on their experience of developing and implementing the in‐house and the commercial automation tools in our institution. Three levels of initial chart check automation were simulated: (1) Auto_UMMS_tool (which consists of in‐house program and commercially available software); (2) Auto_TG275 (with full and partial automation as indicated in TG‐275); and (3) Auto_UMMS_exp (with full and partial automation as determined by our experts’ re‐evaluation).

**Results:**

With no automation of initial chart checks, the ranges of manual check times were 29–56 min (full TG‐315 list) and 102–163 min (full TG‐275 list), which varied significantly with physicists but varied little at different tumor sites. The 69 of 71 checks which were considered as “not fully automated” in TG‐275 were re‐evaluated with more automation feasibility. Compared to no automation, the higher levels of automation yielded a great reduction in both manual check times (by 44%–98%) and potentially residual detectable errors (by 15–85%).

**Conclusion:**

The initial chart check automation greatly improves the practicality and efficiency of implementing the new TG recommendations. Revisiting the TG reports with new technology/practice updates may help develop and utilize more automation clinically.

## 1 INTRODUCTION

Initial chart check, a key component of medical physicists’ clinical responsibilities, has been one of the most effective ways of ensuring compliance with the prescription.^1^ Because of little available guidance on plan and chart review offered by American Association of Physicists in Medicine (AAPM) Task Group (TG) Report 40 and the American College of Radiology (ACR)/American Society for Radiation Oncology,^2^, ^3^ two AAPM TG reports (275^4^ and 315^5^) provide recommendations for clinical practice in terms of chart review in departments of radiation oncology.

TG‐275,^4^ “Strategies for Effective Physics Plan and Chart Review in Radiation Therapy,” was published in January 2020. This report is based on a survey of AAPM’s membership and included 103 multiple‐choice items answered by 1,526 respondents from community hospitals, academic affiliates, and others. The goal of TG‐275 is to establish a baseline for the physics plan and chart review and thereby enhance the safety and quality of care for patients. This TG considers external‐beam radiotherapy (EBRT) with photons, electrons, and proton radiotherapy. A risk‐informed approach considering failure mode and effects analysis (FMEA) was taken to develop the recommendations. For photon/electron EBRT initial plan/chart review checks, 151 check items were proposed under the categories of patient assessment, simulation, and treatment planning. TG‐275 was revised in response to the comments from the full AAPM membership. The authors of this report emphasized that checklists were included only as examples, not as definitive lists for any one clinic.

TG‐315 has been released to the public as a draft of “Plan and Chart Review in External‐Beam Radiotherapy and Brachytherapy” before its publication. Unlike TG‐275, the goal of TG‐315 is to provide essential checklist items according to the minimum practice standards for EBRT and brachytherapy. Supported by the ACR, TG‐315 excludes some TG‐275 recommendations that were deemed beyond the clinical training and responsibility of a medical physicist. In its public review version, the TG‐315 draft proposes a checklist with 44 items for EBRT. These items are categorized into 36 recommended items and 8 optional items. The optional items can be added to the checklist depending on specific practices at each institution. TG‐315 also recommends that each institution establishes its local standard format for treatment prescription.

Current initial chart checks still rely heavily on human inspection and evaluation of various aspects of treatment plans. However, many studies have called for the improvement of pretreatment physics review performance by introducing initial chart check automation. For example, to quantify the potential effectiveness of different quality control measures, some studies have used departmental incident learning systems,^6^ which involve reporting any near‐misses and incidents that occur in the practice of radiation oncology. A study conducted at the University of Washington Medical Center (Seattle, WA) documented 522 potentially severe or critical near‐miss events within an institution‐wide incident learning system over 3 years.^7^ The majority of errors that were not detected could have been identified if automation of specific physics checks had been in place.

Concerning the increasing reliance on initial chart check automation, TG‐275 provides an estimation of the types of checks that might be automated in the future, based on a review of prior publications up to 2016^8^, ^9^, ^10^, ^11^, ^12^, ^13^, ^14^, ^15^, ^16^, ^17^, ^18^, ^19^ and other considerations. The feasibility of automation as indicated in TG‐275, however, may be increasing with the rapid development of techniques in locally developed programs,^20^, ^21^ vendor solutions, and recent acceleration of machine learning efforts.^22^, ^23^, ^24^, ^25^, ^26^, ^27^, ^28^, ^29^, ^30^ Some items previously deemed impossible for automation may become feasible through the development of new and easy‐to‐implement machine learning techniques. One example is a study by Luk et al.^31^ who, using a Bayesian network‐based radiotherapy plan verification model, suggested a 4‐year training dataset to optimize the performance of the network, and yearly updates were considered sufficient to capture the evolution of clinical practice and maintain fidelity. Other recently published papers describe and quantify time savings and error reduction using different analyses.^21^, ^32^, ^33^, ^34^ A review of these new techniques and publications is instructive in looking at the future of initial chart check automation.

To date, no quantitative analyses on the time needed for such chart checks have been provided that take into account the TG‐275 and TG‐315 recommendations. The level of automation that will be required to implement these recommendations in clinical practice without being burdensome is unclear. Here, we explore the future development of initial chart check automation for photon/electron external beam radiation therapy with practical and efficient implementation of the new TG reports. We also estimate, using quantitative analysis, the benefits of time saved and errors avoided by introducing automation consistent with the new TG reports.

## 2 METHODS

### 2.A Evaluation of manual initial chart check time for different scenarios

An in‐house study was conducted to evaluate manual initial chart check time for the full checklists of TG‐315 and TG‐275. The TG‐315 full checklist refers to the entire list of Table 4 in the public review version of the TG‐315 draft. For the minimum acceptable safety standards, particularly for the technical component, the initial treatment plan EBRT checklist in TG‐315 contains 44 items for physicists. The TG‐275 full checklist refers to all items listed in its Table S1A.ii^4^. This full list includes more than 150 check items under the categories of patient assessment, simulation, and treatment planning for photon/electron EBRT initial plan/chart review checks. Eight ABR‐certified medical physicists in our department with differing clinical experience (2–20 years) were invited to participate in a study based on their clinical experience. Manual initial chart check times were evaluated for six different tumor sites (prostate, whole pelvis, lung, breast, head and neck, and brain cancers) based on each physicist’s experience. Three types of plans — intensity‐modulated radiation therapy (IMRT)/volumetric‐modulated arc therapy (VMAT), 3D, and simple calculation — were evaluated (depending on their applicability in each cancer site).

Two derived checklists were created for TG‐315 and TG‐275 to eliminate some uncommon check items for different scenarios. In this article, “TG‐315 recommended checklist” refers only to the 36 recommended items in Table 4 of TG‐315. For TG‐275, a priority checklist was created based on the highest risk priority number (RPN) of the corresponding failure modes (FM) and use frequency. Ninety‐five items with RPN > 100 and use > 60% were included in the priority checklist.

Therefore, four checklists were involved in our data analysis: (1) the TG‐315 recommended checklist; (2) the TG‐315 full checklist; (3) the TG‐275 priority checklist; and (4) the TG‐275 full checklist. Our physicists’ experience in the Department of Radiation Oncology at University of Maryland Medical System (UMMS) sites was based on using Varian ARIA as the radiation oncology information system (OIS), Raysearch RayStation as the treatment planning system (TPS), and Varian C‐series and TrueBeam linac machines for EBRT treatment delivery.

### 2.B Re‐evaluation of automation feasibility of TG‐275 checklists

Our expert development team including three physicists re‐evaluated the TG‐275 automation feasibility. This expert team has been developing automation tools for various EBRT procedures in our institution during the past 7 years. Our in‐house automation tool for initial chart check includes the sophisticated scripts that can access electronic documents, treatment plan DICOM files, and record and verify (R&V) system. Table 1 shows the functions that were available at the time of publication for this in‐house tool, which includes many items in the TG‐275 checklist. Besides, a commercial tool — Mobius3D (Varian; Palo Alto, CA) has been used in our initial chart check procedures for years. Our experience with our in‐house and commercial tools helps classify the feasibility of automating TG‐275 items. Note, to be consistent with TG‐275, full automation refers to “can potentially be fully automated” and partial automation refers to “can potentially automate whether particular information is present (e.g., a document exists) but not whether the information in it is correct.”

**TABLE 1:** Five categories of automated initial chart check items covered by our current in‐house tool (=Auto_UMMS_Tool excluding the commercial tool) that has been used clinically for years. The corresponding TG‐275 checklist items of patient assessment (PA), simulation (Sim), and treatment planning (TP) in Table S1.A.ii are also listed.

UMMS tool check items	Corresponding TG‐275 items in Table S1.A.ii
Prescription	
Prescription consistency with our institutional practice guidelines	PA‐Q1‐1, PA‐Q1‐9, Sim‐Q1‐2, TP‐Q2a‐10
Prescription template name, creation date, approval status and physician attestation	PA‐Q1‐2, TP‐Q6‐5
Pregnancy/waiver check, previous treatment history, chemotherapy, cardiac devices check	PA‐Q1‐5, PA‐Q1‐6, PA‐Q1‐7, PA‐Q1‐8
Diagnosis code and description check. Consistency between treatment site and diagnosis code. Check diagnosis documents	PA‐Q1‐3, PA‐Q1‐5, TP‐Q2a‐1, TP‐Q2a‐2
Check additional documents (such as patient survey/consent)	PA‐Q1‐11, PA‐Q1‐13
Technique and energy consistent with plan	TP‐Q2a‐4, TP‐Q2a‐11, TP‐Q2a‐12
Plan compliance. For example, no high‐energy plan for patients with cardiac device	TP‐Q3a‐3
Prescription approval	PA‐Q1‐2
Total dose = fraction dose × fraction number	TP‐Q2a‐3,6,7,8
Image technique	TP‐Q8‐5,8
Fractionation, such as, BID	TP‐Q2a‐13
Setup comments	Sim‐Q1‐1,9
Bolus (thickness, type, use frequency)	TP‐Q2a‐5
Image and contour	
Check simulation order document	Sim‐Q1‐1, Sim‐Q1‐12
Check simulation summary document	Sim‐Q1‐3, Sim‐Q1‐4, Sim‐Q1‐5, Sim‐Q1‐8, Sim‐Q1‐9
Check CT DICOM files	Sim‐Q1‐10, Sim‐Q1‐12, Sim‐Q1‐13, Sim‐Q1‐14, Sim‐Q2‐1
Check CT series description	Sim‐Q1‐14, Sim‐Q2‐2
CT protocol	Sim‐Q1‐10
CT density table	TP‐Q4a‐7
CT contrast	Sim‐Q1‐14
Max HU	TP‐Q1a‐6
CT modification	Sim‐Qa‐11,12
BB position consistent with user origin and localization point	Sim‐Q1‐6, Sim‐Q1‐7
Density override	TP‐Q1a‐7, TP‐Q4a‐6
CT thickness for different planning techniques	Sim‐Q1‐18
Contours	TP‐Q1a‐1,2,3,4,7
Check high‐Z materials in CT and max HU outside/inside External	TP‐Q1a‐6
Structure approval	TP‐Q1a‐8
Check treatment couch insertion and other structures	TP‐Q1a‐9
Beam and plan	
Patient treatment position	Sim‐Q1‐15
Beam naming	TP‐Q6‐4
Plan deliverability, such as max/min MU for different techniques, max/min spot weight	TP‐Q6‐1,2
Electron block	Sim‐Q1‐3,4, TP‐Q6‐8,13, TP‐Q7a‐15
Total body irradiation (TBI) parameters (TBI insert, dose rate, etc.)	PA‐Q1‐6
Special requirements for stereotactic radiosurgery (SRS), stereotactic body radiation therapy (SBRT)	TP‐Q4a‐3,4
Dose calculation algorithms/engines	Sim‐Q1‐6
Mono‐isocenter check	Sim‐Q1‐6,7, TP‐Q6‐18
Beam isocenter relative to the target center	TP‐Q6‐17
Dose grid relative to external	TP‐Q4a‐5
Setup beams consistent with image techniques in the prescription	TP‐Q6‐1,4
For SRS, cone and multileaf collimator (MLC) plan choice based on target size	TP‐Q2a‐12, TP‐Q6‐1,3
For SRS, gap between jaw and MLC leaf	TP‐Q7a‐13,14,15,16
For SRS, check MRI image and image registration between MRI and CT	TP‐Q10‐1,2
Dose grid size for different plans	TP‐Q4a‐5
Check motion management techniques	Sim‐Q2‐3
Check optimization parameters and settings	TP‐Q1a‐5, TP‐Q4a‐1, TP‐Q4a‐2,3,4
Check isocenter shifts for multiple iso‐center plans	TP‐Q3a‐1, TP‐Q3a‐2
Plan quality	
Target coverage (CTV and planning target volume [PTV])	TP‐Q5‐1,2,3,4,5,6,7
Contour expansion from CTV to PTV	TP‐Q1q‐4
Max dose point	TP‐Q5‐5
Plan quality index (such as, DVH, conformity index, gradient index, homogeneity index, etc.)	PA‐Q1‐10, TP‐Q5‐3
Dose distribution for dose greater than 110% of total prescription dose	TP‐Q5‐5, TP‐Q5‐7
ARIA	
Plan setup in ARIA	TP‐Q7a‐1,6 to TP‐Q7a‐24,27
Fractionation consistent with prescription	TP‐Q7a‐2,3,4,5
Plan scheduling	TP‐Q10‐15
Dose limits (session/daily/total) consistent with prescription	TP‐Q7a‐25,26
Course openness	TP‐Q7a‐28
Image (kV and cone‐beam Ct) approval	TP‐Q8‐2 to TP‐Q8‐8
Course name consistent with prescription template name	TP‐Q6‐5
Treatment journal entries	TP‐Q7a‐21
Delta couch. SRS requires delta couch. Other plans (photon/electron) require delta couch cleared	TP‐Q6‐18
Reference dose points	TP‐Q7a‐3,4,5,10
Tolerance tables for different plans, such as SRS.	TP‐Q6‐14, TP‐Q7a‐20
Check peer review task in ARIA carepath	PA‐Q1‐12
Check billing approval in ARIA carepath	PA‐Q1‐14
Check setup note in ARIA	Sim‐Q1‐9
Compare DICOM files (CT, RT‐Structure, RT‐Plan) from TPS and ARIA	Sim‐Q1‐16, all items in TP‐Q7a
Beam arrangement vs standard plan templates	TP‐Q6‐1

### 2.C Automation level simulation for initial chart check

In this work, three levels of chart check automation, that is, Auto_UMMS_tool, Auto_TG275, and Auto_UMMS_exp, were simulated for the four checklists. Auto_UMMS_tool refers to the automation level that automates some checklist items by using our in‐house tool (Table 1) and Mobius3D. Auto_TG275 refers to the automation level that automates some checklist items fully or partially as indicated in TG‐275 Table S1. A.ii^4^. Auto_UMMS_exp refers to the automation level that allows fully or partially automated checklist items as re‐evaluated by our expert development team.

Our UMMS tool is composed of the in‐house automation tool and Mobius3D, and both use DICOM files for CT image, RT structures, RT plan, and RT dose as input data. The in‐house tool was designed to automatically review the items in Table 1. It compares all plan parameters in a DICOM RT‐Plan file from the OIS to those in its counterpart DICOM RT‐Plan file from the TPS. A comparison PDF report can be generated as a patient EMR (electronic medical record) document. In the report, the hospital name, patient name, ID, plan Name/Label, and approval status in TPS and ARIA OIS are listed. Any difference in monitor units (MUs), multileaf collimator (MLC) shape, energy, collimator angle, gantry angle, gantry rotation, couch angle, source–skin distance jaw position, isocenter, segment weights, wedge, bolus, patient position, or applicator can be highlighted if that difference exceeds the predefined tolerance. For the majority of plan parameters, the predefined tolerance is zero. Nonzero predefined tolerance for some plan parameters is mainly due to rounding errors while importing/exporting plans between different systems. More information, including plan name, beam name, radiation type, tolerance table, isocenter coordinate, and treatment machine name, is also compared. The commercial software Mobius3D, powered by its own Mobius Calculation module with a GPU‐accelerated collapsed‐cone dose algorithm, recomputes the 3D dose distribution on the planning CT from TPS and then compares the dose distribution from its engine against the dose distribution from TPS on the same planning CT. In the Mobius QA reports, the mean dose and D95 are presented for all the target structures. The 3D global gamma and the point dose at the dose specification point for each beam are calculated in Mobius and compared against TPS, Mobius also provide other information such as beam deliverability.

The potential for automation of chart check items was mentioned in TG‐275 Table S1. A.ii^4^. Some check items are regarded as potentially fully automated, including physician intent/prescription vs treatment plan), optimization or calculation parameter checks (target and organ at risk objectives, algorithms, dose grid size, etc.), and data transfer from the TPS to a third‐party OIS. For some items, automation may be possible only to determine whether a specific document or item is present, not whether the information in that item is correct (e.g., most patient assessment and simulation checks). The remaining items require manual inspection. Most of them are related to free‐typing or handwriting documents, such as consult note, physics consult, patient consent documents.

### 2.D Benefit evaluation

Benefit evaluation for different automation levels was performed based on two aspects: (1) manual check time saving and (2) avoidance of errors as a result of automation.

For time‐saving estimation, a weighting score was assigned to each check item to scale the reduced manual check time corresponding to each of the three automation levels: “Full” = 0, “Partial” = 0.5, and “No” = 1. For a check item with automation level between any two of them, the weighting score was averaged. For example, if a check item was determined as “Full/Partial,” the automatic weighting score for the manual check time was (0 + 0.5)/2 = 0.25.

For the avoidance of errors as a result of automation, results from Gopan et al.^7^ Table 3 were used for an estimation. Gopan et. al provided the percentage of potentially detectable errors for each step in the radiation therapy process, including patient assessment, simulation, and treatment planning. Here, we defined "the residual detectable errors" as the detectable errors that require human intervention due to the limited automation. We expected that introducing more automation can catch more detectable errors automatically, yielding fewer residual detectable errors. To approximately estimate the relationship between residual detectable errors and chart check automation, we assumed that the number of residual detectable errors for the manual check is proportional to the number of the checklist items that cannot be automated.

## 3 RESULTS

Manual initial chart check time that was averaged over six tumor sites varied among different physicists’ responses in this study. Table 2 shows that the maximum, the median, and the minimum manual check time values scattered across a large range. Such differences were not correlated with the physicists’ experience; instead, differences grew as planning techniques became more complex. Note that some check items were considered to be manually impractical (e.g., MLC control points) and were excluded when our physicists estimate their check time for this study, so the actual time to finish the corresponding checklist took even longer. Different tumor sites have little impact on manual check time for each physicist: the manual check time difference for different tumor sites ranged from −3% to 4% of their average value. These time data were normalized to the average value to eliminate physicist‐specific difference.

**TABLE 2:** The maximum, the median, and the minimum average manual inspection time values for six tumor sites estimated by eight physicists with differing clinical experience to manually finish TG‐315 and TG‐275 checklists. No specific relationship was found between time and experience, but manual check time can vary significantly among physicists.

	Average manual check time over six tumor sites (min)		
	IMRT/VMAT	3D	Simple calc
TG‐315 full list			
Max	66.5	55.5	50.5
Median	50.3	44.6	19.1
Min	8.8	9.2	9.9
TG‐275 full list			
Max	185.5	133.0	95.0
Median	72.8	71.8	37.1
Min	36.5	34.6	16.0
TG‐315 recommended list			
Max	51.9	47.7	36.9
Median	42.2	37.6	17.0
Min	6.1	6.6	7.8
TG‐275 priority list (UF > 60%)			
Max	144.6	91.1	62.6
Median	48.1	47.1	21.0
Min	23.0	22.5	3.5

The automation feasibility of chart check items mentioned in TG‐275 Table S1. A.ii^4^ was a result of the survey from the AAPM members. Here, our expert development team re‐evaluated the automation feasibility based on our experience of developing and implementing our in‐house automation tool and the commercial product across our institution. Among the 71 items that were deemed not fully automated in the TG‐275 report, the automation feasibility of 69 checks was re‐evaluated differently. Table 3 lists some of our results versus TG‐275: 35 items as “Full”, 17 as “Full/Partial”, 6 as “Partial”, 2 as “No”. The additional feasibility option “Full/Partial” means that automation can be partially implemented but full automation can be realized with certain conditions. For example, our institution is still using a scanned patient consent form. If an electronically fillable or online patient consent form is used, all the essential information could be retrieved by our in‐house tool. However, using an electronically fillable or online patient consent form requires our current clinical procedure and policy to be altered, which may take a long process. Therefore, the feasibility for “patient consent” in Table 3 was re‐evaluated as “Full/Partial” given the fact that we can only verify if the consent document exists and accessing the content of the document may be possible in the future.

**TABLE 3:** An example of our expert team’s re‐evaluation vs TG‐275 survey results on the automatic feasibility of EBRT initial chart check items in TG275. The automation feasibility was categorized as “Full,” “Full/Partial,” “Partial,” or “No.” The re‐evaluation was based on the existing automation tools Auto_UMMS_Tool (that is already being used clinically) plus those scripts and programs that are under development by our expert development team.

Photon categories	Photon physics check	TG‐275	UMMS experts’ re‐evaluation
Patient assessment	Prescription (with respect to standard of care or institutional clinical guidelines)	No	Full
	Diagnosis definition, including imaging and outside records	No	Full/partial
	Pathology report	No	Partial
	Medical chart to confirm laterality, site, etc.	No	Full/partial
	Special considerations for radiotherapy (e.g., pacemakers, ICDs, pumps, etc.)	Partial	Full
	Previous radiotherapy treatments	Partial	Full/partial
	Utilization of other treatment modalities (i.e., chemotherapy, surgery)	No	Full/partial
	Patient consent	Partial	Full/partial
	Peer review of treatment decision (e.g., tumor board, peer‐to‐peer evaluation, etc.)	Partial	Full
	Consult note	Partial	Full/partial
	Insurance approval	Partial	Full
Simulation	Physician directive for imaging technique, setup and immobilization (may include contrast, scanning orientation, immobilization device, etc.)	Partial	Full
	Description of target location on physician planning directive (e.g., RUL Lung, H&N, L1‐L4)	Partial	Full
	Utilization of immobilization and ancillary devices	No	Full
	Construction of immobilization and ancillary devices	No	No
	Written or photographic documentation of patient positioning, immobilization, and ancillary devices	Partial	Full
	Isocenter placement	No	Full/partial
	Isocenter consistency between patient marking and setup instructions	Partial	Full
	Patient setup and positioning	Partial	Partial
	Setup note	No	Full/partial
	CT scanner technique (e.g., kV, filter, etc.)	No	Full
	CT scan artifacts	No	Partial
	CT scanning range (i.e., superior–inferior range includes entire target and organs at risk [OARs])	No	Full/partial
	CT scan field of view and clipping of anatomy	No	Full/partial
	Use of contrast and corresponding effects on HU number	No	Partial
Items reviewed that are part of motion management	4D CT parameters and data set	No	Full
	Breath‐hold parameters and dataset	No	Partial
	Gating parameters	No	Partial
Treatment planning contouring checks: items reviewed during contour checks:	Target(s)	Partial	Full
	OARs	Partial	Full/partial
	Body/external contour (if required/applicable)	Partial	Full/partial
	High‐Z material, contrast, artifacts	No	Full/partial
Treatment planning prescription checks (physician intent/Rx vs treatment plan): Items reviewed for prescription checks:	Additional shielding	No	No
	Prescription vs consult note	No	Full
Dose distribution and overall quality of the plan	Dose distribution	No	Full
	Prior radiation	Partial	Full
	Plan sum (e.g., original plus boost plans)	No	Full
Standard operating procedures of practice followed or correctly used	Setup note	Partial	Full
	Field aperture	No	Full
	Setup shifts	No	Full
	Treatment plan warnings/errors	Partial	Full/partial
Data transfer from TPS to a third‐party OIS (e.g., Eclipse to MOSAIQ, Pinnacle to ARIA, etc.)	Couch parameters	Partial	Full
	Setup note	Partial	Full
	Imaging sequence	Partial	Full
Setup for image guidance for treatment planning	Matching instructions (e.g., 2D/2D, 3D, etc.)	No	Full
	Imaging technique	No	Full
	DRR image quality	No	Full
	Matching structures	Partial	Full
During a patient’s treatment course, verify that the original plan and corresponding dosimetry (i.e., DVH, target coverage, OAR sparing, etc.) still meet the treatment intent by using the original plan on a new simulation CT set	Old/new CT registration	No	Full
	Isocenter placement	No	Full
	Deformed or new contours	No	Full
Other checks during the initial plan check process	Registration/fusion of image sets (CT, PET, MRI, etc.)	No	Full/partial
	Image set chosen for treatment planning	No	Full
	Physician‐designed apertures	No	Full/partial
	Physics consult (e.g., evaluation of dose to pacemaker, previous treatment, etc.)	No	Full
	Parameters and setup for specialized devices (e.g., ExacTrac, VisionRT, RPM, etc.)	No	Full
	Request for in vivo dosimetry	Partial	Full
	Motion management instructions	Partial	Full/partial
	Instruction for replanning	No	Full
	Scheduling of tasks (e.g., weekly chart checks, MD image review, etc.)	No	Full

The automation feasibility of each chart check item relies on the availability to retrieve the medical information as well as the availability of high‐level automation software, either in‐house or commercial. Below we present several examples that illustrate the differences between the results of our re‐evaluation and the TG‐275 survey results.

**FIG. 1:**
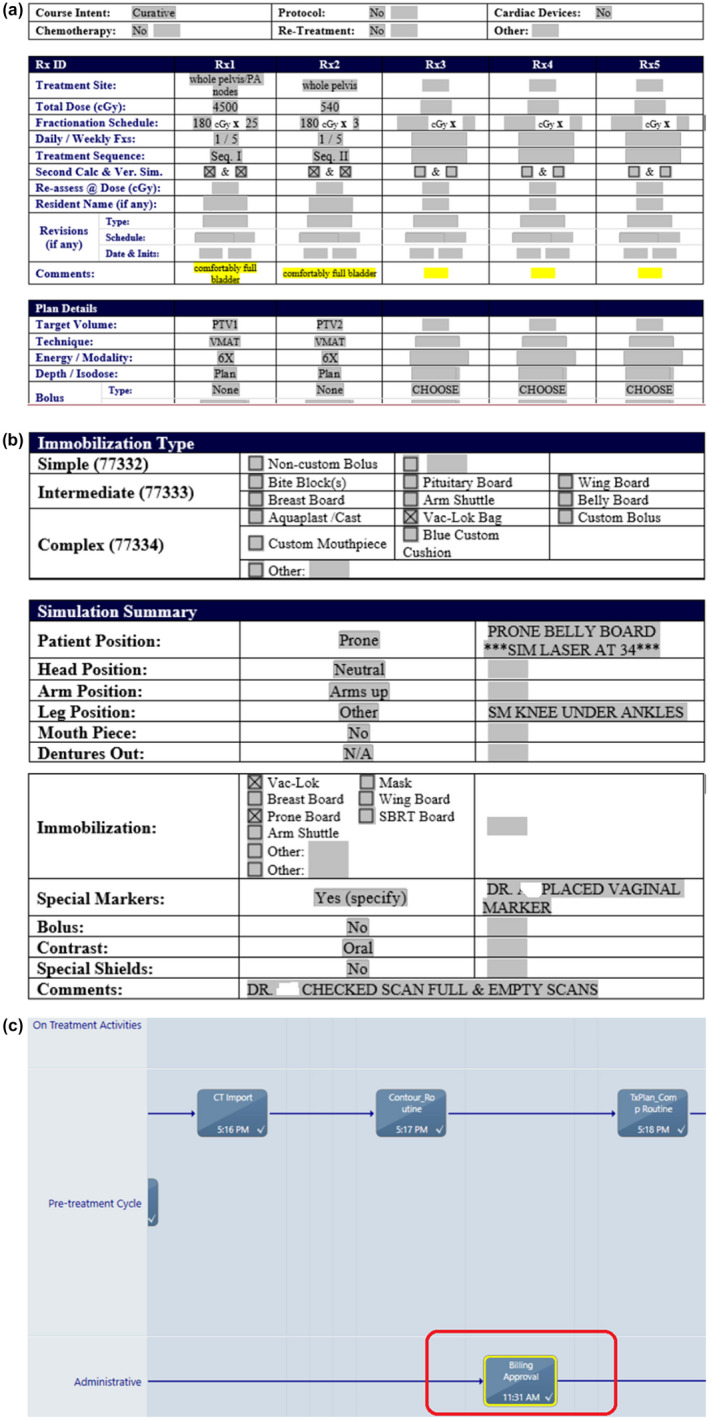
(a) A snapshot of part of prescription document for a pelvis patient in our institution, (b) a snapshot of part of the Simulation Summary document in our institution, and (c) a snapshot of part of the Varian ARIA’s Care Path with billing approval.

One example is about the “Prescription (with respect to standard of care or institutional clinical guidelines)”. TG‐275 deemed its automation as “No” while our team considered it “Full.” Our institution uses a set of Microsoft Word templates for the prescription documentation in the Varian ARIA‐based EMR system. The use of these electronic documents (as opposed to the scanned paper documents with handwriting in some other clinics) allows our scripts to access the content of a specific document and compare its items with the treatment plan and the ARIA databases. Our automation tool first queries and retrieves each patient’s prescription information based on the ARIA patient ID, treatment site, and document template name. Then, the tool parses all the prescription information as shown in [Fig. 1(a)] (The readers are referred to a recently published paper^35^ for more details.). Meanwhile, our institution has produced a series of clinical practice guidelines for prescribing, simulation, contouring, planning, and evaluation of EBRT for each stage of various diseases. As the standard of our clinical practice, these guidelines are periodically updated by our radiation oncology teams based on their clinical experience and the latest literature publishing. Our automation tool compares an individual prescription against our corresponding practice guideline by using an xml template for the essential information, therefore the item “Prescription (with respect to standard of care or institutional clinical guidelines)” of TG‐275 is already automated in our institution, which explains why our team unanimously thought it should be considered “Full.” The access to the contents of such electronic documents and our practice guidelines allows us to propose different automation feasibility of many items in Table 3 like “Prescription (respect to standard of care or institutional clinical guidelines,” “Utilization of immobilization and ancillary devices” [Fig. 1(b)], “Special Considerations for radiotherapy,” and “Utilization of other treatment modalities,” “Request for in vivo dosimetry,” and “Parameters and setup for specialized devices.” Another example is about “Insurance approval.” Our automation tool queries the Varian ARIA SQL database to check if the care path task “Billing Approval” is completed by the billing office [Fig. 1(c)] and verify if the scanned insurance card document exists. Our automation tool can also analyze the treatment plan details by accessing the planning system DICOM files, such as isocenter positioning with respect to the target, the CT image properties, and the plan quality, which also yield more advanced automation feasibility compared to the TG‐275 survey results.

**FIG. 2:**
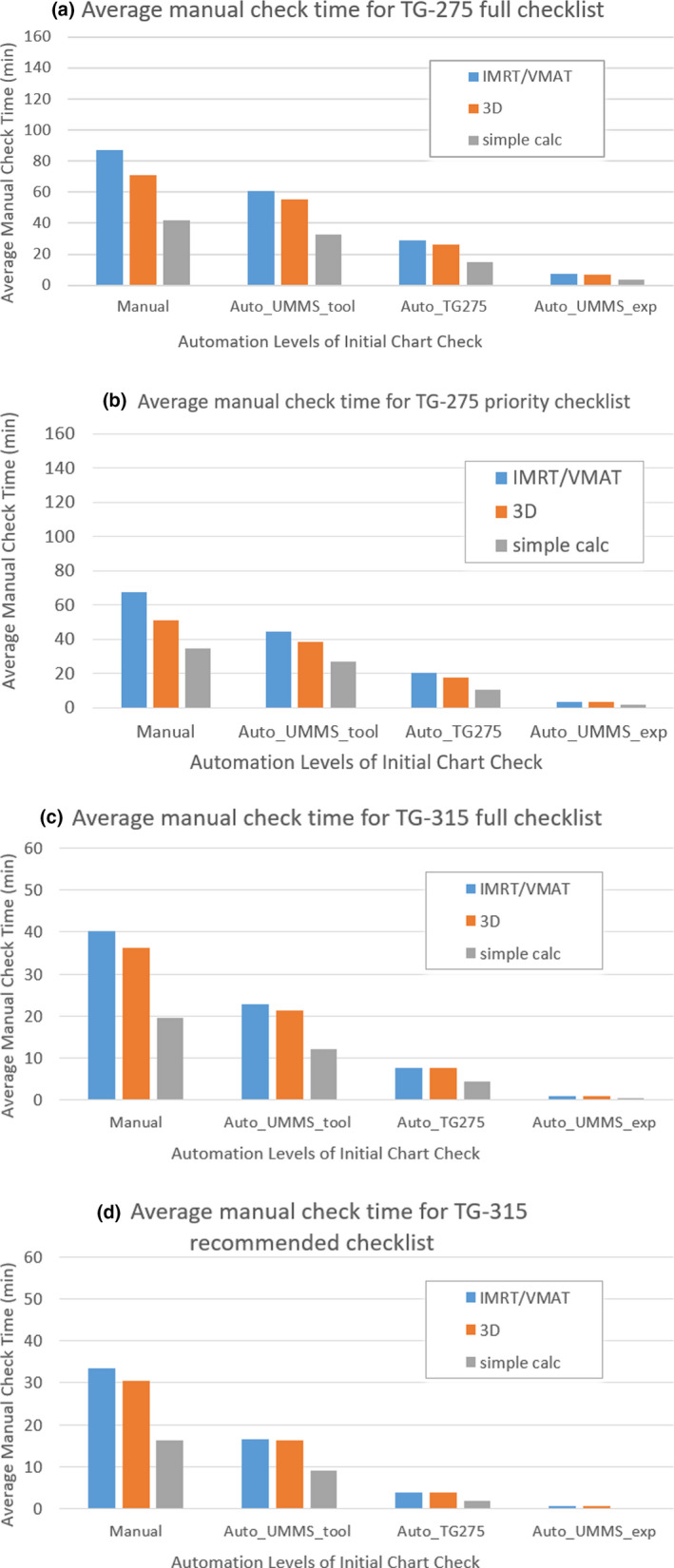
Residual manual check time (averaged for all physicists) for different automation levels when using the (a) TG‐275 full checklist; (b) TG‐275 priority checklist; (c) TG‐315 full checklist; or (d) TG‐315 recommended checklist.

As more automation is introduced, manual time can be reduced significantly. Data in Fig. 2 indicate that as higher level automation is introduced, manual check times can be greatly shortened. Using the Auto_ UMMS‐tool, Auto_TG275, and Auto_UMMS_exp automation levels for the full TG‐275 checklist, residual manual check times with no automation were reduced by about 30%, 67%, and 91%, respectively, for IMRT/VMAT; by 22%, 63%, and 91%, respectively, for 3D; and by 22%, 64%, and 91%, respectively, for 2D simple. Time reduction in percentage for different checklists was quite similar. For the TG‐275 priority checklist using the Auto_UMMS‐tool, Auto_TG275, and Auto_UMMS_exp automation levels, manual check times were reduced by 34%, 70%, and 95%, respectively, for IMRT/VMAT; by 22%, 66%, and 94%, respectively, for 3D; and by 22%, 69%, and 94%, respectively, for 2D simple. For the TG‐315 checklists, up to 50%, 81%, and 98% of the time can be saved from pure manual check by using the Auto_UMMS‐tool, Auto_TG275, and Auto_UMMS_exp automation levels, respectively. If the Auto_UMMS_exp automation level is achieved, the residual manual check time is shortened to < 10 min for the TG‐275 checklists and < 1 min for the TG‐315 checklists.

Auto_TG275 can help reduce time by 3%, 7%, and 35% for the patient assessment, simulation, and treatment planning steps, respectively. Auto_UMMS_Exp can help to achieve 6%, 18%, and 46% in corresponding reductions.

Table 4 details the reductions in the potentially residual detectable events in different radiation workflow steps for automation levels Auto_TG275 and Auto_UMMS_Exp. Data from Gopan et al.^7^ were used to estimate this benefit.

**TABLE 4:** The benefit of reduction in residual potentially detectable issues for the manual check when using full TG‐275 checklist and automation levels Auto_TG275 and Auto_UMMS_Exp.

Workflow step	% of potentially detectable events originating at this step^7^	% of residual detectable event due to Auto_TG‐275 level of initial chart check	% of residual detectable event due to Auto_UMMS_Exp level of initial chart check
Patient assessment	7.7	4.6	1.5
Simulation	28.2	21.1	9.6
Treatment planning	49.2	13.7	3.4

## 4 DISCUSSION

Since the physics check is one of the most effective checks for radiation treatment,^1^ it is essential to stay up to date on the latest recommendations from new guidelines. For TG‐315 and TG‐275, which led to discussions about standardizing initial chart checks and the potential inclusion of automation, a careful review is needed to determine the potential for future clinical workflow improvement. This article explores the development of initial chart check automation and assesses the potential benefits as part of implementing the new guideline in routine clinical practice. Although our automation experience is institution‐specific, our method can be adapted to other institutions and our work will provide an instructional reference to those who are interested in realizing their automation potential in the initial chart check procedure.

While TG‐275 provides an overview of published studies on automatic checking (Section 2. D.) and suggestions for software vendors (Section 5. E.), it also addresses its limitations (Section 6). For example, TG‐275 states that “It is the hope of this Task Group that this report and the data in it will be revisited as technologies and practices evolve” and “the impact of these recommendations has not been carefully studied, since this is beyond the scope of the charges of the Task Group.” The new results presented in our work show additional automation beyond TG‐275. We intended to offer a quantitative reference not only for physicists but also for developers who are interested in revisiting TG‐275 with the new updates of technologies or practices. The updates may vary with different institutions, but we believe such variation will be less and less significant with more prevalence of automation tools.

The results suggest a strong need for the development of automated initial chart checks for the sake of efficiency and efficacy. Introducing a high level of initial chart check automation may be the best solution to significantly ease the human workload and reduce human error. This is particularly important as our treatment techniques become more complex within the framework of precision radiotherapy. We believe that by introducing automation tools into initial chart checks for different levels of errors, from simple to sophisticated can be rapidly detected without human manual inspection. Regardless of the automation level used, we believe that human vigilance is always needed, particularly when it comes to the prevention of a medical event.

Check items in TG‐275 that were considered beyond the clinical training and responsibility of a medical physicist as in TG‐315 could be re‐examined when we are equipped with automated tools. According to the International Organization for Medical Physics’ Policy Statement No. 1,^36^ it is physicist's obligation to supervise QA programs and optimization of therapeutic procedures. This does not exclude cross‐check of the work done by radiation oncologists, dosimetrists, therapists, or other radiation oncology team members. Once an automation tool is clinically implemented, each user must fully understand its limitations and outputs. Lack of such understanding might lead to adverse consequences in patient care.

In assessing the status of automation tool development, it seems likely that lower dimensional problems, such as treatment parameter comparison, can be easily handled by scripts/programs. Higher dimensional problems in physician order error, including disease staging and treatment modality decision, may be taken care of by machine learning, such as a k‐means clustering algorithm,^8^ random forest methods,^37^ or Bayesian networks as proposed by Kalet et al.^38^ and further developed by Luk et al.^31^ As Kalet et al.^39^ and Pallai et al.^40^ pointed out, machine learning still faces many challenges and must be quality assured before introduction into the clinic. The breakthrough of automation tools or machine learning beyond low‐level checks will take some time.

A limitation of this report is in quantitative analysis; our study data from physicists may be biased by their familiarity with current checklists and hardware/software, as well as by nonfamiliarity with the new checklists. After becoming accustomed to the new checklists, physicists may spend less time on TG‐315 or TG‐275 items. However, we believe our results can provide insight into the process of evolving current initial chart check procedures to be consistent with the latest national guideline. While TG‐315 is very similar to the current checklist in many clinics, it may add extra safety to also include most of the recommended items in TG‐275. As pointed out in TG‐275, items suggested in the report may be applied after considering each institution’s workload. More automation tools will boost initial chart check efficiency, and then, it becomes practically feasible to include more check items suggested by TG‐275.

## 5 CONCLUSION

Without automated initial chart checks, the implementation of new guidelines, particularly TG‐275, involves significant human work. Automated initial chart checks can significantly reduce manual check time and detect more potential errors. With the evolution of automation techniques, it is foreseeable that more automated checks will be available to further improve practicality and efficiency in the clinical implementation of the new TG recommendations. Revisiting the TG reports with new technology and practice updates may help develop and utilize more potential automation for clinical use.

## Data Availability

The data that support the findings of this study are available from the corresponding author upon reasonable request.
